# Genetic, Immunological, and Public Health Perspectives on Podoconiosis

**DOI:** 10.1155/jotm/9961827

**Published:** 2025-11-25

**Authors:** Tsegahun Asfaw Abebe, Abiola Isawumi

**Affiliations:** ^1^West African Centre for Cell Biology of Infectious Pathogen, University of Ghana, Volta Road, Accra LG 54, Ghana; ^2^Department of Biochemistry, Cell and Molecular Biology, University of Ghana, Volta Road, Accra LG 54, Ghana

**Keywords:** genetic susceptibility, immunological mechanisms, neglected tropical diseases, podoconiosis, public health

## Abstract

Podoconiosis is a neglected tropical disease (NTD) that primarily affects poor communities in tropical regions, particularly in sub-Saharan African countries. The disease results from prolonged contact with red clay soils, leading to chronic lymphedema of the lower limbs. However, the pathogenesis is not yet fully clarified, which continues to hinder the development of targeted interventions and definitive diagnostic tools. This review synthesizes recent advancements in understanding the genetic, immunological, and tissue-level factors driving podoconiosis to help bridge this knowledge gap. It also addresses the clinical features, epidemiology, and public health impact of podoconiosis, focusing on the challenges of misdiagnosis and the lack of standardized diagnostic tests. The identification of biomarkers for early detection and the development of targeted interventions are critical steps toward mitigating the economic and social burden of podoconiosis. Comprehensive research into the genetic and immunopathological basis of podoconiosis is essential for advancing diagnostic and therapeutic approaches and supporting global eradication efforts.

## 1. Introduction

Neglected tropical diseases (NTDs) are caused by infectious agents in the tropical and subtropical regions. They are often associated with poverty, inequality, and environmental factors [1] and cause serious public health problems with socioeconomic consequences. They are described as “neglected” as they are underrepresented in the global health scheme and under-researched with limited funding [1]. Podoconiosis has recently gained global attention following its inclusion in the WHO's Skin NTD strategy. This recognition facilitates integrated care pathways with other NTDs, such as lymphatic filariasis, and promotes a more coordinated approach to management and control. It is one of NTD's priority diseases in Ethiopia, Uganda, Rwanda, and Cameroon, and it has been included in their long-term health strategies [2]. In endemic countries, it poses a significant but often ignored threat to social, economic, and public health burdens.

Podoconiosis has been reported in regions of tropical Africa, South America, Southeast Asia, and the Indian subcontinent [1]. It is more common than HIV, malaria, and tuberculosis in endemic areas where people do not wear shoes regularly and encounter red clay soil [3]. Clay soil contains minerals such as crystalline silica, aluminosilicate, stacked kaolinite, iron oxide, and zirconium, which enter the lymphatic system via the skin, causing inflammation and swelling of the lower limbs [4]. The disease is characterized by lymphedema, warty lesions, plantar nodules, and deformity [5].

Podoconiosis has an economic, psychological, social, and physical burden on affected individuals [6]. For example, in Ethiopia, it has a higher economic burden than NTDs like trachoma, onchocerciasis, and leishmaniasis [7]. Despite its significant burden, a coordinated global elimination strategy is absent. This stands in contrast to national-level efforts, such as the National Strategy for Podoconiosis in Ethiopia [8]. The lack of a worldwide strategy could be due to either lack of awareness or underestimation of the burden. The absence of a simple, point-of-care diagnostic test remains a major obstacle. While clinical diagnostic algorithms have been developed and validated in Ethiopia [9], the reliance on clinical exclusion challenges accurate disease mapping and almost certainly leads to an underestimation of the true incidence and prevalence of podoconiosis. Furthermore, the disease is little known to healthcare professionals or health systems and is often confused with lymphatic filariasis [10].

Despite progress in podoconiosis research, the incomplete understanding of the molecular mechanism underlies a major barrier to the innovation of diagnostic and therapeutic intervention [11]. This gap in knowledge hinders the identification of biomarkers associated with increased risk, specific immune dysregulation, and histopathological changes contributing to disease progression. This review summarizes recent insights into the pathogenesis of podoconiosis and discusses their implications for improving disease diagnosis, treatment, and public health control.

### 1.1. Rationale for the Review

Poor people in tropical highlands frequently walk barefoot, which increases the risk of exposure to irritating clay soil particles [12]. When prolonged exposure is combined with a hereditary predisposition, the lymphatic capillaries become damaged, leading to the swelling of the lower legs over time [4]. Although consistent footwear use from childhood prevents podoconiosis, this is often impractical in endemic areas due to widespread poverty. It is also extremely logistically difficult to provide and distribute strong shoes in a sustainable manner due to the large number of risk populations in endemic countries [13]. For instance, there are an estimated 43.8% of Ethiopians who are at risk and reside in rural areas [14]. Despite having shoes, farmers still prefer to work in the clay soil barefoot because it is slippery and sticky.

Regardless of the commitment to mitigate public health problems in podoconiosis-endemic countries, there is no comprehensive strategy for control or elimination. The lack of reliable data on podoconiosis creates significant challenges in making informed decisions about resource allocations, healthcare workforce training, program monitoring, research priorities, policy advocacy, and health system integration. The disease mechanisms, including immunogenetics and pathogenesis, that lead to pathological changes are not fully understood. Therefore, studying podoconiosis, including genetic, immunological, and tissue features, will inform the development of diagnostic tools, effective interventions, and control strategies.

## 2. Methods

This review was conducted through a literature search of databases including PubMed, Scopus, Web of Science, and Google Scholar. Additional sources like the WHO report, government health department publications, and relevant gray literature were also included. A combination of keywords, including *podoconiosis, epidemiology, genetic susceptibility, immunological mechanisms, neglected tropical disease, lymphedema, HLA class II, public health burden, diagnosis,* and *treatment*, was used to identify relevant articles. Boolean operators (AND, OR) were used to refine the search. The search was not restricted by publication date to ensure the capture of foundational historical studies on podoconiosis, given its long-standing but neglected nature. Key studies were critically analyzed to synthesize current knowledge, identify knowledge gaps, and propose future directions. The referenced database was built with EndNote (Version 21.5), and information was summarized into tables, figures, and paragraphs.

## 3. Result and Discussion

### 3.1. History and Global Epidemiology of Podoconiosis

Podoconiosis is derived from the Greek words “podos” (foot) and “konos” (dust). It is a nonfilarial, chronic geochemical lymphedema of the lower extremities that is now known to affect barefoot people, such as farmers, living primarily in areas with red clay soil [15]. By the late 19^th^ century, a discrepancy in the distribution of lymphedema and filaria in North Africa, Central America, and Europe was observed. In 1938, Robles [5] made the first clear distinction in Guatemala, identifying individuals with lymphedema who tested negative for bacteria and microfilariae, linking the condition to barefoot walking. Then Oomen [16] associated nonfilarial lymphedema with high altitudes, while Price's later studies described the disease's bilateral leg swelling and skin changes, coining the term podoconiosis [17–20].

Podoconiosis has been reported in at least 33 countries worldwide. However, its prevalence is highly heterogeneous [1]. It is a significant public health problem with well-documented high prevalence in specific regions of countries like Ethiopia, Rwanda, and Cameroon. In contrast, in many countries, including South America and Southeast Asia, reports are historical or anecdotal, and the true prevalence remains unknown, likely to represent scattered cases rather than a common burden [1]. In Africa, the disease has been reported in Burundi [20], Cameroon [21], Cape Verde [22], Equatorial Guinea [23], Ethiopia [24], Kenya [25], Rwanda [26], Sao Tome and Principe [27], Sudan [23], Tanzania [28], and Uganda [29]. Podoconiosis has also been reported in the Central American highlands in Ecuador, Mexico, Guatemala, Brazil, Suriname, and French Guiana on the South American coast [30, 31]. In Asia, podoconiosis has also been reported in northwestern India [32], Sri Lanka, and Indonesia [30, 31].

The highest prevalence of podoconiosis was reported in Tanzania, Kenya, Uganda, Ethiopia, and Cameroon (Table 1). Among the affected countries, an estimated 1.5 million affected people are in Ethiopia (more than 25% of the global total) [3]. In Ethiopia, irritating clay soil covers around 24% of the entire land area, where 43.8% of the population lives [14]. Depending on the amount of red clay soil present, the prevalence of podoconiosis in endemic areas of Ethiopia ranges from 2.8% to 15% [38, 39]. In Africa, 114.5 million people are living in areas suitable for podoconiosis [13].

### 3.2. Pathogenesis and Clinical Features of Podoconiosis

**Figure 1:**
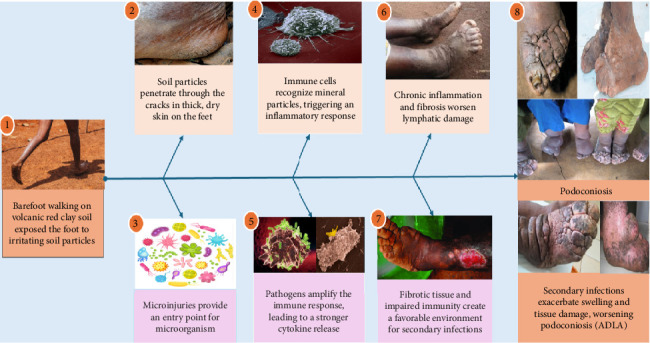
Immunopathological mechanism of podoconiosis with secondary infection: (1) Initial exposure to mineral particles, (2) mineral particles from volcanic soil penetrate skin, causing microinjuries, (3) microorganisms may enter through the damaged skin, (4) macrophages recognize mineral particles and pathogens, triggering an inflammatory response, (5) T-cells and B-cells are activated, amplifying cytokine release, (6) chronic inflammation leads to fibrosis and lymphatic damage, (7) secondary infection arises due to impaired immunity and fibrotic tissue, and (8) clinical manifestations include swelling, skin ulcers, and signs of infections (figure designed by authors with pictures randomly sourced from Google, a public query for common use).

Prolonged exposure coupled with a genetic predisposition to the disease causes lymphatic damage and swelling of the lower limbs over time [15]. Based on available data, mineral particles induce inflammation in people with a genetic predisposition that leads to lymphedema. An inflammatory response is triggered by the interplay of genetics and different environmental factors, which results in lymphatic fibrosis [40]. Although the mechanism is unclear, the mineral particles engulfed by macrophages cause an inflammatory reaction in the blood vessels, resulting in fibrosis, edema, and severe elephantiasis [4] (Figure 1). It was demonstrated that minerals entering through the skin are engulfed by macrophages and reach lymph nodes, where they produce an inflammatory reaction [37]. For example, silicate particles induce subendothelial edema, endothelial inflammation, and collagen deposition [40]. Individuals with podoconiosis also exhibited thicker dermal collagen, diminished elastic fibers, dilated blood vessels, scattered macrophages, and cellular infiltration [41].

Podoconiosis is a result of human leukocyte antigen (HLA)–mediated immunological response [11]. The HLA class II region is implicated in immune responses, suggesting that abnormal immune activation may contribute to the pathogenesis of podoconiosis [42]. Additionally, elevation of inflammatory transcripts in podoconiosis patients suggests podoconiosis is a result of chronic immune activation [43] (Figure 1). However, the distinct immunological mechanisms and downstream signaling pathways involved in the uptake of particles, the onset of inflammation, and finally the tissue damage leading to lymphedema are still unknown.

Clinical symptoms of podoconiosis usually begin with frequent burning and itching sensations in the foot [43, 44]. Late podoconiosis is characterized by interdigital fusion and ankylosis of the interphalangeal and ankle joints [15]. The disease is bilateral but asymmetrical and is most often confined to the below-knee body parts. Podoconiosis symptoms, including acute adenolymphangitis (ADLA), are thought to result from bacteria entering cracked foot skin and triggering inflammation [45, 46] (Figure 1). But again, it is still unclear if pathogens like bacteria, fungi, or viruses play a role during the onset and development of podoconiosis.

### 3.3. Risk Factors of Podoconiosis

**Figure 2:**
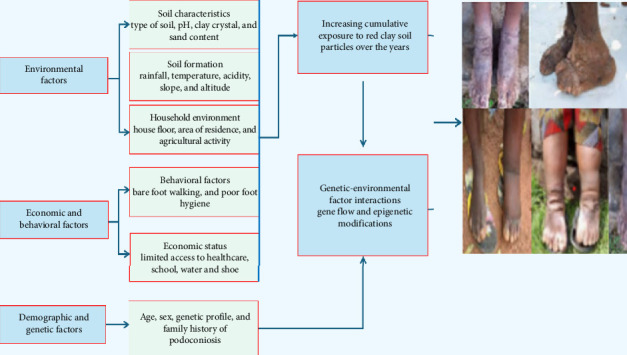
Podoconiosis conceptual framework showing interrelatedness of different risk factors (figure designed by the authors with pictures randomly sourced from Google, a public query for common use).

Podoconiosis is a complex disease influenced by genetics, environmental, geographical, socioeconomic, and cultural factors [47] (Figure 2). The family clustering of podoconiosis indicates that genetic elements are possible predisposing factors, with an estimated sibling recurrence rate (λs) and heritability of 5.05 and 0.63, respectively [47]. Furthermore, single nucleotide polymorphisms (SNPs) in the HLA class II region (DRB1, DQA1, and DQB1) have a substantial correlation with podoconiosis [48, 49]. Gene ontology analysis revealed that differentially expressed genes in patients with podoconiosis were closely related to genes associated with MHC protein, cytokines, and TNF receptors [42]. In addition, people with podoconiosis were characterized by lower hydration of the stratum corneum of the skin of the legs and feet than healthy people [15]. Therefore, they are at higher risk of developing podoconiosis due to cracking and splitting of the skin, which may be the entry point for soil components and pathogens (Figure 1).

The specific cause of podoconiosis in red clay soils remains uncertain. Little research has focused on the relationship between the major risk factors and podoconiosis. For example, the specific component(s)/molecule(s) within the volcanic red clay soil in combination with altitude, rainfall, and temperature remain unknown. However, epidemiological and observational research indicates that various environmental elements (soil geochemical and mineralogical features) are linked to podoconiosis. For instance, a recent study revealed high concentrations of phyllosilicate clays (smectite and kaolinite), mica groups, quartz (crystalline silica), iron oxide, and zirconium have been described in high-prevalence areas of podoconiosis [4].

Podoconiosis is also significantly associated with various sociodemographic and behavioral factors (Figure 2). For example, in the Ethiopia wealth index, the habit of not wearing shoes (from a young age), lack of foot hygiene, remoteness of medical facilities, family history of leg swelling, and large family size were identified as risk factors [12]. Podoconiosis has also been associated with age and gender, with women at high risk in Ethiopia [4]. It often starts between the ages of 10 and 19, with many cases (87%) occurring in the economically productive age range (15–65 years) [50].

### 3.4. Public Health Burden of Podoconiosis

The prevalence and burden of podoconiosis may be underestimated due to its misclassification as other diseases (lymphatic filariasis and leprosy). However, podoconiosis imposes significant economic burdens, as most affected individuals are in productive working-age groups [50]. It also causes economic losses of more than $208 million annually in Ethiopia. The national economic burden of podoconiosis is much higher than other NTDs in Ethiopia, like trachoma, onchocerciasis, and leishmaniasis [7]. Due to frequent complications, stigma, and discrimination, victims are forced to skip work or reduce their working hours, which negatively impacts their social, economic, and psychological well-being. Furthermore, it contributes to significant prejudice in schools, workplaces, and markets, as well as isolation from social bonds such as marriage [51].

The most prevalent consequence of podoconiosis is complication, as patients scored significantly lower on all quality-of-life parameters (hygiene, diet/nutrition, and access to shoes). These situations therefore lead to ADLA, which is one of the main outcomes and complications of podoconiosis, resulting in a 24- to 149.5-working-day loss of activity per year [47]. Cracked skin on the feet allows bacterial entry, triggering ADLA episodes [45, 46]. These recurrent infections drive inflammation and lymphatic damage. The growing threat of antimicrobial resistance (AMR) complicates treatment, as resistant bacteria prolong or worsen ADLA [46], hindering effective management of podoconiosis and increasing the disease burden.

### 3.5. Current Diagnosis and Control Strategies of Podoconiosis

Accurate and precise diagnosis of podoconiosis is critical for patient safety, research, and surveillance. However, to date there is no point-of-care screening test and standard diagnostic tool for podoconiosis. Nowadays, podoconiosis is a diagnosis of clinical symptoms and exclusion of other forms of lymphedema based on the patient's history, physical examination, and disease-specific diagnostics (Table 2). This can frequently result in misdiagnosis, as the most common differential diagnoses for podoconiosis include lymphatic filariasis, leprosy, and rheumatic lymphedema.

So far, the major strategies to control podoconiosis include preventing contact with irritating soils and treating lymphedema morbidity [57]. Wearing footwear and socks, practicing regular good foot care, and covering the floor at home are all primary preventative methods for podoconiosis. Foot cleansing and care, including regular moisturizing to manage cracked feet and reduce the risk of secondary infection, wound care, compression, leg exercise and elevation, treatment of secondary infections, and cleaning shoes and socks are also important to prevent progression of the disease [1]. But widespread implementation is hindered by poverty, lack of education, and limited access to healthcare. Additionally, there is no global elimination strategy, and the role of AMR in managing secondary infections like ADLA is an emerging challenge.

### 3.6. Disease Mechanisms and Future Perspectives

#### 3.6.1. Understanding Susceptibility

Genetic factors are crucial in determining why certain individuals develop podoconiosis despite being exposed to the same environmental conditions as others who remain unaffected [47]. While prolonged exposure to irritant minerals in red clay soil is the primary environmental trigger, not all individuals who are exposed develop the disease [17]. Specific genetic markers, particularly within the HLA class II region, are strongly associated with an increased risk of developing the disease [48, 49]. The HLA class II genes play a critical role in regulating the immune system's response to foreign substances (e.g., silica), and variations in these genes can lead to an abnormal immune response when exposed to soil minerals [42]. In individuals with podoconiosis, it is thought that these genetic variants lead to chronic inflammation in the lymphatic system, causing lymphedema.

While widespread genetic screening in resource-limited settings is currently impractical, identifying genetic markers provides crucial insight into disease pathogenesis. In the long term, if a cost-effective genetic test were developed (e.g., targeting a key HLA haplotype via PCR), it could be important to stratify risk within already prioritized communities. This would allow for the most efficient targeting of existing preventative interventions (e.g., footwear distribution) to those at highest genetic risk, maximizing the impact of limited resources. The primary intervention must remain the widespread dissemination of footwear and education. Understanding the genetic predisposition to podoconiosis also opens the door for future therapeutic developments, where interventions could be tailored to modulate the immune response in genetically susceptible individuals. Ultimately, integrating genetic data into public health strategies could significantly enhance the efficacy of disease prevention and control in podoconiosis-endemic regions.

#### 3.6.2. Clarifying Immune Response

The immunological mechanisms involving chronic inflammation and lymphatic damage in podoconiosis remain unclear. Podoconiosis occurs when individuals are exposed to irritant mineral particles in volcanic red clay soil, which are thought to penetrate the skin and enter the lymphatic system, triggering a prolonged immune response [40]. In individuals with a genetic predisposition, this immune activation becomes chronic, leading to inflammation, fibrosis, and eventually lymphedema [42]. The role of immune dysregulation in podoconiosis has been supported by findings of increased activation of T-cells, classical monocyte, and dendritic cell markers [11]. However, the exact pathway through which the immune system responds to these environmental triggers is still unclear.

Over 40% of podoconiosis patients showed significant changes in some hematological parameters like plateletcrit (PCT), platelet distribution width (PDW), mean platelet volume (MPV), mean cell volume (MCV), and red cell width (RCW) [58]. A decrease in absolute neutrophil counts also suggests a possible link to an autoimmune process [59], calling for further mechanistic study. Hemoglobin level also showed a reduction in podoconiosis patients [58], suggesting activation of proinflammatory cytokines suppresses erythropoietin response to anemia and shortens red blood cell (RBC) half-life [60]. The other possible cause might be due to the release of inflammatory proteins (hepcidin and lactoferrin) [60]. Oxidative stress markers, including peroxides, antioxidants, superoxide dismutase, and growth factors, were also identified as a potential mechanism of podoconiosis [61] but lacked immunological data to clarify the disease mechanism and progression.

One key area of focus is the role of macrophages, which are immune cells that engulf foreign particles. In podoconiosis patients, macrophages are believed to engulf soil particles that enter through the skin [40]. These macrophages may then migrate to the lymph nodes, where they initiate an inflammatory cascade, resulting in the release of proinflammatory cytokines and the recruitment of additional immune cells. This chronic inflammatory response contributes to fibrosis and structural damage observed in the lymphatic system of podoconiosis patients. Moreover, immune system genes, particularly within the HLA class II region, have been implicated in the abnormal immune activation of the disease [42], suggesting that certain genetic variants may enhance susceptibility by amplifying immune responses to environmental irritants.

Studying the immune response in podoconiosis is critical not only for understanding the pathogenesis of the disease but also for developing therapeutic interventions. By identifying the specific immune pathways involved, researchers can target these pathways to develop treatments that modulate immune activity. For example, therapies that reduce the overactive immune response or prevent macrophage activation may help mitigate the chronic inflammation and tissue damage that characterizes podoconiosis. Furthermore, understanding the immune mechanisms may help identify biomarkers for early detection, allowing for interventions before significant lymphatic damage occurs. Thus, a detailed exploration of the immunological processes in podoconiosis can provide essential insights for both the prevention and treatment of the disease.

#### 3.6.3. Improving Diagnosis

The diagnosis of podoconiosis currently relies on clinical exclusion, where physicians rule out other causes of lymphedema, such as lymphatic filariasis and leprosy, based on patient history, physical examination, and disease-specific tests [62]. This approach often leads to delayed or inaccurate diagnosis, especially in resource-limited settings where access to healthcare and diagnostic tools is minimal. A deeper understanding of the genetic and immunological factors involved in podoconiosis can significantly improve diagnostic accuracy by identifying molecular biomarkers specific to the disease.

Despite the limited number of molecular studies, primarily from Ethiopia, emerging research has identified promising biomarker candidates for podoconiosis. These include genetic susceptibility markers (e.g., HLA-DRB1∗0701, DQA1∗0201, DQB1∗0202) [48, 49], elevated serum levels of proinflammatory cytokines (e.g., TNF-α, IL-1β, and IL-6) indicating chronic immune activation [11, 59], and a distinct transcriptomic signature involving upregulation of MHC class II and inflammatory genes [42]. The development of a point-of-care test based on these biomarkers, such as a lateral flow assay or a minimally invasive genetic test, could revolutionize disease management. These molecular markers can be detected through blood tests or skin biopsies, providing a quicker and more accurate diagnosis, enabling earlier intervention, and preventing the progression of the disease. Early diagnosis through molecular biomarkers would not only reduce the physical and economic burden on patients but also help healthcare systems better allocate resources.

#### 3.6.4. Guiding Treatment Development

Gaining a deeper understanding of the tissue-level changes, particularly lymphatic fibrosis and chronic inflammation, is essential for developing more effective therapeutic interventions. The current management of podoconiosis focuses largely on symptom relief, such as foot hygiene, bandaging, and elevation to manage swelling, and antibiotics to treat secondary infections [1]. While these measures are crucial, they do not address the underlying immunological and fibrotic pathology.

The elucidated mechanisms, such as chronic immune activation mediated by HLA class II variants and the ensuing proinflammatory cytokine release (e.g., TNF-α, IL-1β, and IL-6) [11, 42, 59], provide a strong rationale for investigating immunomodulatory therapies. Similarly, the extensive collagen deposition and lymphatic fibrosis [41] suggest antifibrotic agents could be beneficial. However, it is important to note that the translation of these mechanisms into specific, approved treatments for podoconiosis represents a fundamental knowledge gap. The nascent state of targeted drug discovery for this disease means that precise therapeutic candidates are not yet available. Rather than speculate on specific drugs, we emphasize that this mechanistic understanding should guide a new research agenda.

Future studies must now prioritize targeted drug repurposing screens to identify existing immunomodulatory or antifibrotic agents (e.g., those used in rheumatoid arthritis or idiopathic pulmonary fibrosis) that could be evaluated for efficacy in podoconiosis. Concurrently, a major effort in biomarker discovery and validation is needed to develop objective tools for diagnosis and monitoring treatment response. By focusing on these priorities, the research community can build the necessary evidence base to move from symptomatic care to targeted therapies that address the core causes of podoconiosis.

#### 3.6.5. Public Health Implications and Policy

Delayed diagnosis and intervention in podoconiosis have significant socioeconomic impacts on affected individuals. Podoconiosis primarily affects people in low-income, rural areas who are often unable to afford shoes [39]. The disease typically manifests in the economically productive years of life, leading to physical disability, loss of mobility, and social isolation [51]. Individuals suffering from podoconiosis often face stigma, limiting their access to education, employment, and social activities [51]. As a result, families of affected individuals experience an increased financial burden due to the inability to work and the cost of care [51]. Furthermore, the lack of widespread knowledge and recognition of disease among healthcare providers often results in misdiagnosis, with podoconiosis being confused with other causes of lymphedema, like lymphatic filariasis, delaying appropriate treatment [63].

Integrating biomarker-based strategies into public health policies can significantly improve the prevention and treatment of podoconiosis in endemic regions. Biomarkers could enable earlier and more accurate diagnosis, reducing the time delay between disease onset and intervention. This would allow patients to receive care at an earlier stage, mitigating the progression of disabling symptoms and reducing the economic burden on families and healthcare systems. In regions with limited resources, the development of simple, cost-effective diagnostic tests based on biomarkers could be implemented at the community level, empowering local health workers to screen and monitor at-risk populations.

From a policy perspective, governments and global health organizations should incorporate podoconiosis into broader NTD control programs. Public health policies could promote the distribution of protective footwear and education on foot hygiene as preventive measures. Additionally, integrating biomarker-based early detection into existing NTD frameworks would enable better resource allocation, improved disease tracking, and more efficient interventions. Targeted policies that combine prevention with early diagnostic tools would not only improve the individual and social impact of podoconiosis but also help reduce healthcare costs associated with managing late-stage disease complications.

## 4. Conclusion

Podoconiosis remains a significant public health burden in endemic regions, disproportionately affecting impoverished communities with limited access to healthcare. While the link between barefoot exposure to irritant soils and disease development is established, critical gaps remain in understanding the molecular mechanisms of pathogenesis.

This review highlights the pivotal role of HLA class II genetic variants in immune activation, which drives chronic inflammation and lymphatic damage in affected individuals. The identification of these genetic markers not only clarifies the basis of disease susceptibility but also opens opportunities for genetic screening in high-risk populations. Moreover, the chronic immune response points to potential biomarkers that could transform current diagnostic practices, which rely on clinical exclusion.

Informed by this understanding, a dual strategy is essential. In the immediate term, public health efforts must prioritize robust, cost-effective primary prevention through footwear and hygiene education. Concurrently, there is an urgent need to develop the promising biomarkers into affordable, point-of-care diagnostic tools for early detection and intervention before irreversible lymphatic damage occurs.

Ultimately, controlling and eradicating podoconiosis requires addressing these gaps through further research into therapeutic interventions aimed at modulating the immune response and preventing fibrosis. A coordinated strategy that combines robust prevention, sensitive early detection, and novel therapeutic development is essential to eliminate podoconiosis.

## Figures and Tables

**Table 1:** Prevalence of podoconiosis in high endemic countries.

Country	Year	Type of study	Prevalence	References
Uganda	2024	^∗^Population-based cross-sectional survey in seven districts	0.3%−7.2% (total cases 187 from 10,023 participants from all districts)	[33]
Kenya	2021	^∗∗^Nationwide population-based cross-sectional survey	0.0%–3.1% (county-level distribution)	[34]
Rwanda	2019	^∗∗∗^Countrywide cross-sectional survey	28·3 per 100,000 people–119·2 per 100,000 people (district level)	[35]
Cameroon	2018	^∗∗∗^Countrywide mapping survey	1.1%–4.9% (district-level distribution)	[36]
Ethiopia	2015	^∗∗∗^Countrywide mapping survey	0.0%–15% (district-level distribution)	[14]
Burundi	1976	^∗∗∗∗^Nationwide market survey	0.99% (61 cases/6156 participants)	[20]
Tanzania	1956	^∗∗∗∗∗^Community-based survey	2.51% (12 cases/475 participants)	[37]

^∗^Seven districts include Gomba, Hoima, Kasese, Nakapiripirit, Rukungiri, Sironko, and Zombo.

^∗∗^48 villages across 24 subcounties in the western, Nyanza, eastern, northeastern, Rift Valley, and coastal regions.

^∗∗∗^Covers the entire geographic area of the country, including all regions, districts, or administrative units.

^∗∗∗∗^Covers the entire population of the country using marketplaces as hubs to recruit participants.

^∗∗∗∗∗^Four villages (Bukoba, Biharamulo, Ngara, and Kibondo Districts) in Tanzania.

**Table 2:** Profiles of podoconiosis in comparison with similarly diagnosed diseases.

Feature	Podoconiosis	Filarial elephantiasis	Leprotic lymphedema	Systemic disease lymphedema
Onset of swelling	Starts in the foot and progresses upwards [1, 52]	Starts in the leg (not necessarily the foot) [52]	Often associated with leprosy symptoms	Various, depending on the systemic cause
Distribution	Bilateral, asymmetric, and usually below the knees [1, 52]	Commonly unilateral, extends above the knee, and often involves the groin [52]	Involve areas affected by leprosy [52]	It depends on the systemic cause
Sensory perception	Intact in toes and forefoot [1]	Intact in toes and forefoot [1]	Reduced or absent due to peripheral nerve damage [52]	Intact unless systemic disease affects nerves
Neurotrophic ulcers	Absent [52]	Absent [52]	May be present due to sensory loss	Absent unless systemic disease causes
Thickened nerves	Absent [52]	Absent [52]	Present (due to leprosy)	Absent
Family history	30%–40% report affected blood relatives [38]	May happen in households due to shared exposure to filariasis [53]	No specific familial pattern	No specific familial pattern
Environmental exposure	Prolonged exposure to red clay soil [1, 4, 5, 54, 55]	Exposure to filariasis-endemic areas [52]	No specific environmental link	No specific environmental link
Other features	Lymphedema confined to lower limbs and no systemic involvement [1]	May have systemic filariasis symptoms and groin involvement [52]	Associated with leprosy skin lesions and nerve damage	Linked to systemic diseases (e.g., heart, kidney)
Diagnostic tests	Clinical history, physical examination, and exclusion of other causes [9]	Antigen-based ICT (e.g., Binax®Now Filariasis ICT), antibody tests [56]	Clinical examination and leprosy tests (AFB stain, molecular and lepromin test)	Systemic evaluation of other organs
Treatment	Regular foot hygiene, compression therapy, elevation, footwear use, and lymphedema management [1]	Antifilarial drugs (diethylcarbamazine [DEC], ivermectin, and albendazole), lymphedema management [52]	Multidrug therapy (dapsone, rifampicin, and clofazimine), nerve protection, and lymphedema care	Treat underlying systemic disease (e.g., heart failure, kidney disease, and rheumatic lymphedema)

## Data Availability

No new data were generated or analyzed in this study.
